# Extension of the taxonomic coverage of the family GH126 outside Firmicutes and in silico characterization of its non-catalytic terminal domains

**DOI:** 10.1007/s13205-020-02415-x

**Published:** 2020-09-08

**Authors:** Lenka Kerényiová, Štefan Janeček

**Affiliations:** 1 Laboratory of Protein Evolution, Institute of Molecular Biology, Slovak Academy of Sciences, 84551 Bratislava, Slovakia; 2 Department of Biology, Faculty of Natural Sciences, University of SS. Cyril and Methodius, 91701 Trnava, Slovakia

**Keywords:** Family GH126, In silico analysis, Bacterial members out-of-firmicutes, Sequence-structural features, Thioredoxin-like fold, Leucine-rich repeat motif

## Abstract

The family GH126 is a family of glycoside hydrolases established in 2011. Officially, in the CAZy database, it counts ~ 1000 sequences originating solely from bacterial phylum Firmicutes. Two members, the proteins CPF_2247 from *Clostridium perfringens* and PssZ from *Listeria monocytogenes* have been characterized as a probable α-amylase and an exopolysaccharide-specific glycosidase, respectively; their three-dimensional structures being also solved as possessing catalytic (α/α)_6_-barrel fold. Previously, based on a detailed in silico analysis, the seven conserved sequence regions (CSRs) were identified for the family along with elucidating basic evolutionary relationships within the family members. The present study represents a continuation study focusing on two particular aims: (1) to find out whether the taxonomic coverage of the family GH126 might be extended outside the Firmicutes and, if positive, to deliver those out-of-Firmicutes proteins with putting them into the context of the family; and (2) to identify the family members containing the N- and/or C-terminal extensions of their polypeptide chain, additional to the catalytic (α/α)_6_-barrel domain, and perform the bioinformatics characterization of the extra domains. The main results could be summarized as follows: (1) 17 bacterial proteins caught by BLAST searches outside Firmicutes (especially from phyla Proteobacteria, Actinobacteria and Bacteroidetes) have been found and convincingly suggested as new family GH126 members; and (2) a thioredoxin-like fold and various leucine-rich repeat motifs identified by Phyre2 structure homology modelling have been recognized as extra domains occurring most frequently in the N-terminal extensions of family GH126 members possessing a modular organization.

**Electronic supplementary material:**

The online version of this article (10.1007/s13205-020-02415-x) contains supplementary material, which is available to authorized users.

## Introduction

In the sequence-based classification of glycoside hydrolases (GHs) of the CAZy database (https://www.cazy.org/; Lombard et al. 2014), the family GH126 was established after the study by Ficko-Blean et al. (2011) delivering the three-dimensional structure and partial characterization as a potential α-amylase of the protein CPF_2247 from *Clostridium perfringens* ATCC 13124 genome (Myers et al. 2006). The structure was solved as that of a typical catalytic (α/α)_6_-barrel fold known, e.g., in the family GH15 glucoamylases (Sauer et al. 2000; Kumar and Satyanarayana 2009; Marin-Navarro and Polaina 2011), but adopted neither by α-amylases from families GH13 and GH57 and even in GH119 (Janecek and Kuchtova 2012; Janecek et al. 2014; Martinovicova and Janecek 2018), nor by β-amylases from family GH14 (Monroe and Storm 2018).

The situation concerning the knowledge of the family GH126 is really intriguing since based on the information available about the family (Lombard et al. 2014), it has not been possible to state definitively whether or not this family can be added to CAZy α-amylase families GH13, GH57 and GH119 established previously (Janecek et al. 2014). Currently (July 2020), the family GH126 counts more than 1000 sequenced bacterial members exclusively from the phylum Firmicutes (Lombard et al. 2014). The three-dimensional structure has been solved for two of them, the above-mentioned CPF_2247 amylolytic enzyme from *C. perfringens* (Ficko-Blean et al. 2011) and the PssZ protein from *Listeria monocytogenes* (Wu et al. 2019). Interestingly, only the former is indicated as biochemically characterized enzyme in CAZy (Lombard et al. 2014); the latter, however, being also partially characterized, i.e., as a glycosidase able to degrade the specific exopolysaccharide of the biofilm matrix consisting of the *N*-acetylmannosamine and galactose in a ratio 2:1 (Koseoglu et al. 2015). It is of note that although the authors of both crucial studies (Koseoglu et al. 2015; Wu et al. 2019) have identified the PssZ protein as the member of the family GH8, the CAZy curators have classified it obviously into the family GH126 (Lombard et al. 2014). The uncertainties of the most appropriate CAZy GH family affiliation of the PssZ protein are understandable, because both the CPF_2247 amylolytic enzyme and PssZ protein exhibit a pronounced structural relatedness not only to family GH8, but also to GH48, with which the GH8 forms the CAZy clan GH-M (Alzari et al. 1996; Parsiegla et al. 1998; Guerin et al. 2002; Guimares et al. 2002). The close structural relatedness concerns also putative active-site residues in GH126 (Ficko-Blean et al. 2011) including the general catalytic acid (Glu84; the CPF_2247 protein numbering). The fact that the members of the clan GH-M (i.e., families GH8 and GH48) represent the inverting β-glucan-active GHs (Henrissat and Davies 1997; Lombard et al. 2014; CAZypedia Consortium 2018) should be carefully considered, especially, if the possibility is taken into account, too, the CPF_2247 amylolytic enzyme may be an α-amylase (Ficko-Blean et al. 2011), i.e., the retaining and α-glucan-active enzyme (Janecek et al. 2014).

With the aim to shed some light on the overall view of the family GH126 and its eventual relationships to other GH families, its detailed in silico analysis has recently been accomplished (Kerenyiova and Janecek 2020), delivering for the first time the seven conserved sequence regions (CSRs) defined for the family as well as its division into two basic evolutionary groups represented by two best studied GH126 members—the CPF_2247 amylolytic enzyme from *C. perfringens* (Ficko-Blean et al. 2011) and the PssZ protein from *L. monocytogenes* (Wu et al. 2019). Amylolytic enzymes are, in general, typical modular GHs, possessing, in addition to catalytic domain, also other modules—some contributing with a characteristic function, some seemingly without playing any special role or with a role not recognized as yet (Kuchtova and Janecek 2016; Da Lage 2017; Valk et al. 2017). Among them, the best known and studied module is represented by starch-binding domains (SBD; Janecek et al. 2011), classified in CAZy among the carbohydrate-binding module (CBM) families (Lombard et al. 2014). Until now, 15 SBD CBM families have already been established in CAZy; some additional ones are obviously waiting to be confirmed experimentally that may potentially define new CBM families (Janecek et al. 2019).

Among the family GH126 members, only a few of its members do possess some extra N- and/or C-terminal extensions of their polypeptide chain, i.e., they are mostly formed just by their catalytic (α/α)_6_-barrel domain (Kerenyiova and Janecek 2020). Moreover, from the taxonomical point of view, the family GH126 is a sole prokaryotic—more specifically—bacterial (i.e., not archaeal) family; its members being originating from the phylum Firmicutes only (Lombard et al. 2014; Kerenyiova and Janecek 2020). The present study was, therefore, performed in an effort: (1) to find out reliably whether or not the taxonomic coverage of the family GH126 can be expanded outside Firmicutes; and (2) to characterize by the in silico approaches involving homology modelling and structure comparison the most typical N- and/or C-terminal sequence extensions observed in some extant family GH126 members. The obtained results could thus add another piece of mosaic into the overall picture of this potential α-amylase family.

## Materials and methods

### Sequence collection and evolutionary analysis

Potential members of the family GH126 originating outside the bacterial phylum Firmicutes have been obtained using the basic protein BLAST search (Altschul et al. 1990; https://blast.ncbi.nlm.nih.gov/). As queries, the amino acid sequences of the CPF_2247 amylolytic enzyme from *C. perfringens* (UniProt accession No.: A0A0H2YP60) and the PssZ protein from *L. monocytogenes* (UniProt accession No.: A0A3D7VE02) were used, the searched databases being limited to: (1) *Bacteria* excluding Firmicutes; (2) *Archaea* only; (3) *Eucarya* only; (4) fungi only; (5) plants only; (6) animals only. Seventeen sequences of interest caught by BLASTs (Table 1) were retrieved from GenBank (Benson et al. 2018) and UniProt (UniProt Consortium 2017) sequence databases. For comparison, this sample of out-of-Firmicutes-originating potential GH126 sequences were completed by the representative set of selected 117 GH126 members used in the previous study (Kerenyiova and Janecek 2020) taken directly from CAZy (Lombard et al. 2014; https://www.cazy.org/). It is worth mentioning that to compare only sequence segments obviously formed the basic catalytic core of the family GH126, i.e. the (α/α)_6_-barrel, three of 117 sequences were truncated from their N-terminus—those from *Clostridium butyricum* (GenBank accession No. APF21752.1; residues 1-146), *Lactobacillus brevis* (GenBank accession No. AYM02277.1; residues 1-947) and *Lactobacillus paraplantarum* (GenBank accession No. ALO03904.1; residues 1-236).

**Table 1:** Seventeen hypothetical proteins outside Firmicutes with clear similarities to GH126

No.^a^	Organism	Phylum	GenBank^b^	UniProt^c^	Length
1	Bacterium BCRC 81,127	Unclassified	WP_135371658.1	UPI00107F4117	379
2	Bacterium BCRC 81,129	Unclassified	WP_135367822.1	UPI00107F44C3	350
3	Bacterium 42_11	Unclassified	KUK13779.1	A0A117KYT7	365
4	*Bacteroides xylanolyticus*	Bacteroidetes	WP_104434259.1	UPI000CEC40E9	538^*e*^
5	*Deltaproteobacteria bacterium*	Proteobacteria	OGQ30614.1	UPI0008C880CE	416
6	*Deltaproteobacteria bacterium*	Proteobacteria	OGQ58036.1	A0A1F9IPT0	437
7	*Mycobacteroides abscessus*	Actinobacteria	CPW32488.1	UPI0001A5C03B	388
8	*Pseudomonas* sp. GW456-E7	Proteobacteria	PNB55453.1	A0A2N8FV32	131^f^
9	*Sphingobacterium faecium*	Bacteroidetes	SJN19201.1	UPI00032F5CEA	388
10	*Synergistetes bacterium*	Synergistetes	HDQ93145.1	---^d^	370
11	*Chlamydia abortus*	Chlamydiae	SHE13947.1	UPI000A27BFEE	364
12	*Klebsiella pneumoniae*	Proteobacteria	OON71423.1	UPI00016B383E	361
13	*Mycobacteroides abscessus*	Actinobacteria	SLB95965.1	UPI0009C51C47	358
14	*Myxococcales bacterium*	Proteobacteria	RJO68936.1	A0A3A4K738	387
15	*Rhizobium* sp. KAs 5–22	Proteobacteria	WP_047792160.1	UPI0006492D13	361
16	*Salmonella enterica*	Proteobacteria	EAU0476096.1	---^d^	281^f^
17	*Salmonella enterica*	Proteobacteria	EAQ6393019.1	---^d^	285^f^

^a^Proteins 1–10 were caught by BLAST with the CPF_2247 protein as the query only; proteins 11–15 were caught by BLAST with both CPF_2247 and PssZ proteins as queries; proteins 16–17 were caught by BLAST with the PssZ protein as the query only. For all 17 proteins, the E-value from all BLAST searches ranged from 6e^−35^ to 4e^−06^, which was considered satisfactory

^b^The accession numbers from the GenBank database

^c^The accession numbers from the UniProt database (UniParc – starting with “UPI”)

^d^The UniProt accession number is still not available

^e^The protein from *Bacteroides xylanolyticus* contains the N-terminal extension (1–154) adopting the thioredoxin-like fold

^f^ Fragment; the sequence does not contain the entire catalytic (α/α)_6_-barrel domain characteristic for the family GH126 that typically covers 7 conserved sequence regions

The final set of 134 sequences was aligned using the program Clustal-Omega (Sievers et al. 2011; https://www.ebi.ac.uk/Tools/msa/clustalo/) with default parameters. The computer-produced alignment was only gently manually adjusted mainly with regard to correct adjustment of seven CSRs.

Two evolutionary trees were prepared: (1) one based on the alignment of the entire sequences with truncating the extra segments from their both N- and C-termini, i.e., just the catalytic GH126 domains were considered; and (2) the other one based on the alignment of seven selected CSRs. Both trees were calculated as maximum-likelihood trees (Jones et al. 1992) using the bootstrapping procedure with 500 bootstrap trials (Felsenstein 1985) implemented in the MEGA software (Kumar et al. 2018; (https://www.megasoftware.net/) applying default programme parameters and the bootstrap. The trees were displayed with the program iTOL (Letunic and Bork 2007; https://itol.embl.de/).

Sequence logos of seven proposed CSRs were created using the online tool WebLogo (Crooks et al. 2004; https://weblogo.threeplusone.com/).

### Homology modelling and structure comparison

Most of the family GH126 members consist of the catalytic (α/α)_6_-barrel fold (Ficko-Blean et al. 2011; Wu et al. 2019), but there are a few GH126 sequences possessing mainly the N-terminal extensions (Kerenyiova and Janecek 2020). In addition to the three members mentioned above (those from *C. butyricum*—GenBank: APF21752.1, *L. brevis*—GenBank: AYM02277.1 and *L. paraplantarum*—GenBank: ALO03904.1), some additional GH126 members have deserved the attention, one of them being extended at the C-terminal end (Table 2). Of the 17 newly identified sequences originating outside the Firmicutes, only 1 from *Bacteroides xylanolyticus* (GenBank accession No. WP_104434259.1; residues 1-154) has been found to possess the extra segment positioned at the N-terminus (Table 1).

**Table 2:** List of ten GH126 proteins possessing either the N- or C-terminal extension

No.^a^	Organism	GenBank^b^	Length	Extension^c^	GH126^d^	Motif^e^	Template (PDB)^f^	CDD^g^	Pfam^h^
1	*Bacillus velezensis*	QHK13041.1	637	458–636	43–353	GGDEF	Signalling protein from *Caulobacter vibrioides* (1W25)	+ +	+ +
2	*Clostridium butyricum*	QJU43754.1	521	36–202	207–520	Trx-like	Protein DipZ from *Mycobacterium tuberculosis* (2HYX)	+ +	+ +
3	*Clostridium butyricum*	APF21752.1	521	53–197	199–520	Trx-like	Protein DipZ from *Mycobacterium tuberculosis* (2HYX)	+ +	+ +
4	*Clostridium butyricum*	AXB84457.1	526	41–207	212–525	Trx-like	Protein DipZ from *Mycobacterium tuberculosis* (2HYX)	+ +	+ +
5	*Heliorestis convoluta*	QGG46501.1	523	~ 1–150	166–520	**---**	No relevant homologous structure found	+ +	DUF
6	*Lactobacillus bifermentans*	QGG60425.1	776	178–294	450–774	LRR	Internalin k from *Listeria monocytogenes* (4L3A)	**---**	+
7	*Lactobacillus brevis*	AYM02277.1	1399	37–759	1051–1390	LRR	Ser/Thr-protein kinase from *Arabidopsis thaliana* (6S6Q)	+ +	+ +
8	*Lactobacillus paraplantarum*	ALO03904.1	658	47–151	317–658	LRR	Internalin k from *Listeria monocytogenes* (4L3A)	**---**	+
9	*Lactobacillus* sp. CBA3606	AVK64614.1	658	47–151	322–658	LRR	Internalin k from *Listeria monocytogenes* (4L3A)	+	**---**
10	*Bacteroides xylanolyticus*	WP_104434259.1	538	36–209	213–532	Trx-like	Protein DipZ from *Mycobacterium tuberculosis* (2HYX)	+ +	+ +

^a^Proteins Nos 1–9 were taken directly from the CAZy database from the family GH126; they all originate from the phylum Firmicutes. The protein No. 1 should belong to the group of the PssZ protein from *L. monocytogenes*, whereas the proteins Nos 2–9 should belong to the group of the CPF_2247 amylolytic enzyme from *C. perfringens* (for details, see Kerenyiova and Janecek 2020). Note, the protein No. 5 from *Heliorestis convoluta* exhibits features of both above-mentioned groups. The protein No. 10 was caught by the BLAST search (cf. Table 1)

^b^The accession numbers from the GenBank database

^c^The borders of individual extensions were decided with respect to: (1) sequence alignment with family GH126 members without any extension (mainly the two members with solved tertiary structure—CPF_2247 and PssZ); and (2) structure homology modelling results obtained by the Phyre2 server

^d^The approximate borders of the family GH126 (α/α)_6_-barrel anticipated from the results provided by the Phyre2 server according to the templates of the CPF_2247 amylolytic enzyme (3REN)

^e^The motifs are abbreviated as follows: GGDEF, a diguanylate cyclase domain with the GGDEF region; Trx-like, thioredoxin-like fold; LRR, leucine-rich repeat

^f^A protein used as one of a few closely related best structural templates for homology modelling by the Phyre2 server (PDB code in parentheses)

^g,h^A search in databases CDD and Pfam using the entire amino acid sequence. The sign “ +  + ” means the results from homology modelling were confirmed. The results were confirmed also for the sign “ + ”; in that case just the first 300 residues from the N-terminal end were used for the particular search. For the protein No. 5: DUF—an archaeal domain of unknown function DUF373 (predicted to be an integral membrane protein with six transmembrane regions)—although shown here, considered irrelevant since spanning only a short region of residues 42–83

All sequence segments additional to the catalytic (α/α)_6_-barrel fold of the family GH126 were modelled using the fold recognition Phyre2 server (Kelley and Sternberg 2009; https://www.sbg.bio.ic.ac.uk/~phyre2/) in an effort to identify their potential structural fold. To confirm and/or complete the homology modelling results, all sequences (Table 2) were also submitted to and cross-validated via the Conserved Domain Database (CDD; Marchler-Bauer et al. 2017; https://www.ncbi.nlm.nih.gov/cdd/) and the Pfam database (El-Gebali et al. 2019; https://pfam.xfam.org/).

The coordinates of all template structures, i.e., the signalling protein from *Caulobacter vibrioides* (Chan et al. 2004), the protein Rv2874 from *Mycobacterium tuberculosis* (Goldstone et al. 2016) and serine/threonine-protein kinase from *Arabidopsis thaliana* (Okuda et al. 2020), were retrieved from the Protein Data Bank (PDB; Berman et al. 2000; https://www.rcsb.org/) under the PDB codes 1W25, 2HYX and 6S6Q, respectively. All structural comparisons were performed using the programme MultiProt (Shatsky et al. 2004; https://bioinfo3d.cs.tau.ac.il/MultiProt/). Structures were displayed by the programme WebLabViewerLite (Molecular Simulations, Inc.).

## Results and discussion

The present study is a direct continuation of the first in silico analysis of the family GH126 published recently (Kerenyiova and Janecek 2020), which delivered the definition of the seven CSRs typical for the family as well as highlighting basic evolutionary relationships within the family together with indicating the relatedness with other GH families in the CAZy classification. Since the family GH126 has been established as a prokaryotic family with all its members originating until now solely from bacterial phylum Firmicutes (Lombard et al. 2014), this study has been conducted to find out if it is possible to extend the taxonomical scope of the family GH126 at least outside the Firmicutes. The second equally important aspect of this study has been evoked by either N- or C-terminal segments of polypeptide chain present in a few family members in addition to their GH126 catalytic (α/α)_6_-barrel domain.

### Taxonomic extension of the family GH126 beyond Firmicutes

To reveal any potential members of the family GH126 outside the phylum Firmicutes, the basic protein BLASTs were executed using the CPF_2247 amylolytic enzyme from *C. perfringens* (Ficko-Blean et al. 2011) and the PssZ protein from *L. monocytogenes* (Wu et al. 2019) as queries. The searches were focused on all taxa excluding Firmicutes and then specifically only on *Archaea*, *Eucarya*, fungi, plants and animals.

Of all sequences caught by the individual BLAST searches, 17 proteins have been identified as relevant family GH126 members outside Firmicutes (Table 1). All of them, however, still rank among *Bacteria*, i.e., no protein either of archaeal or eukaryotic origin has been found as potentially belonging to the family GH126. Whereas some of them are characterized as “unclassified bacteria”, most originate from the phylum Proteobacteria, additional phyla such as Actinobacteria, Bacteroidetes and Chlamydiae being also represented (Table 1).

**Fig. 1:**
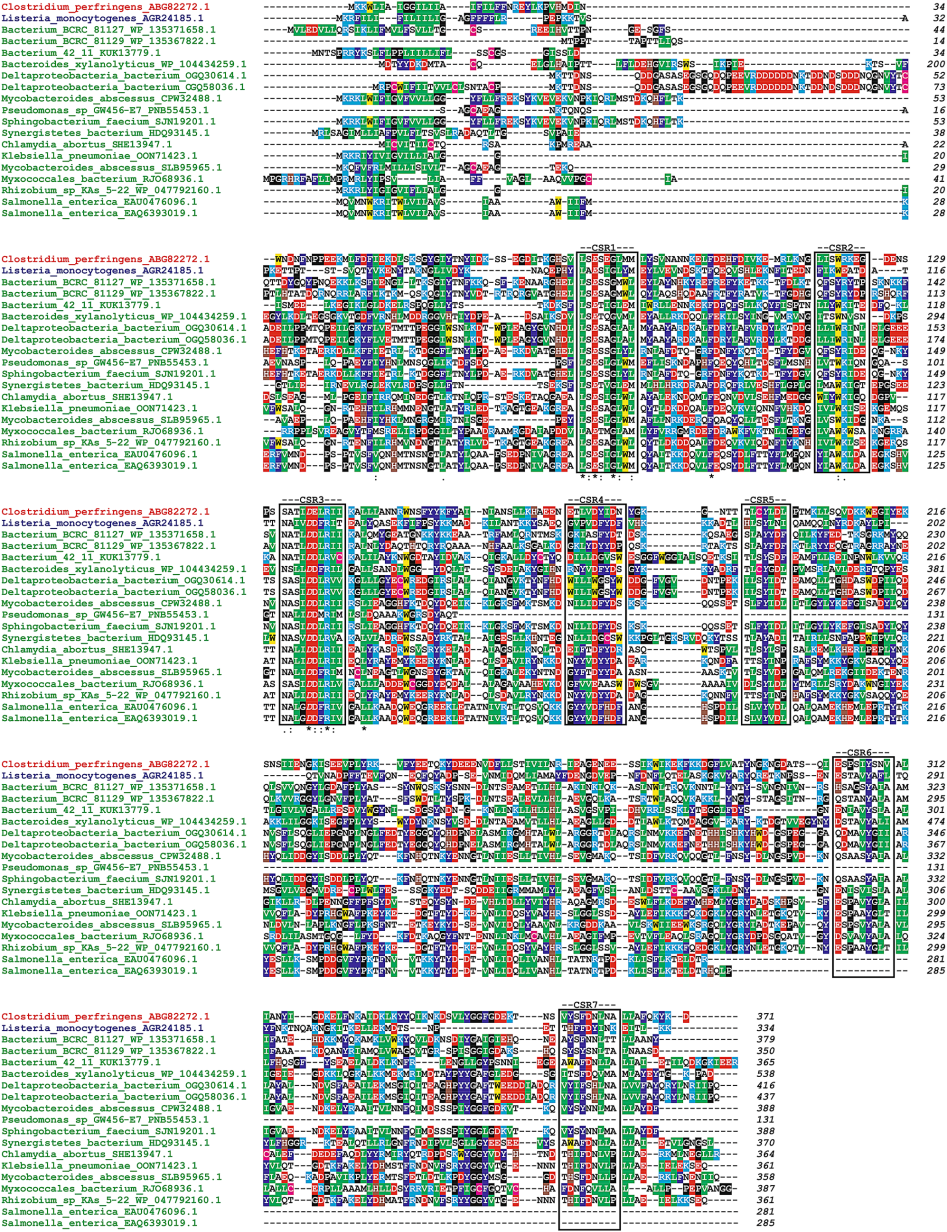
Sequence alignment of potential members of the family GH126 originating outside the phylum Firmicutes with two best studied family representatives. Seventeen putative family members (cf. Table 1) are shown in green, while the two representatives of the family GH126, the CPF_2247 amylolytic enzyme from *C. perfringens* and the PssZ protein from *L. monocytogenes*, are coloured red and blue, respectively. Note, the N-terminal extension (residues 1-154) of the protein from *Bacteroides xylanolyticus* has been cut off as well as the protein from *Pseudomonas* sp. GW457-E7 and both from *Salmonella enterica* represent fragments with respective lengths of 131, 281 and 285 residues, respectively. The seven conserved sequence regions characteristic for the family GH126 (Kerenyiova and Janecek 2020) are boxed and indicated above the alignment. The two potential catalytic residues—Glu84 in CSR-1 and Asp136 in CSR-3 (CPF_2247 numbering) as well as the potentially functional aromatics—Tyr194 in CSR-5 are italicized. Identical positions and conservative substitutions are signified by asterisks and dots/colons, respectively, under the alignment. The colour code for the selected residues: W, yellow; F, Y—blue; V, L, I—green; D, E—red; R, K—cyan; H—brown; C—magenta; G, P—black

The potential affiliation of these proteins to family GH126 has been based on the presence of CSRs in their sequences (Fig. 1) including the invariant residues (Kerenyiova and Janecek 2020), i.e., potential catalytic machinery—the CPF_2247 protein numbering—Glu84 (CSR-1) and Asp136 (CSR-3), functional Tyr194 (CSR-5) plus the Arg139 (CSR-3) and Tyr307 (CSR-6). It is worth mentioning that while the former three residues are present also in sequences of members of the clan GH-M, i.e., families GH8 and GH48 (Ficko-Blean et al. 2011), the latter two are unique for the family GH126 (Kerenyiova and Janecek 2020). Three of the 17 identified proteins (Table 1) are, however, fragments or obviously contain an incomplete (α/α)_6_-barrel domain characteristic for the family GH126, such as the protein from *Pseudomonas* sp. GW456-E7 (GenBank accession No.: PNB55453.1) and the two ones both from *Salmonella enterica* (GenBank accession Nos: EAU0476096.1 and EAQ6393019.1) lacking the C-terminal segments starting closely before the CSR-4 and CSR-6, respectively (Fig. 1). It is of note that the additional tyrosine mentioned above (Tyr307 in CSR-6) is not conserved in the sequence of the protein from *Synergistetes bacterium* (GenBank accession No.: HDQ93145.1). On the other hand, the reliability of the affiliation of all 17 proteins to the family GH126 is strongly supported by the fact that the structure of the family GH126 representatives, i.e., the CPF_2247 amylolytic enzyme from *C.perfringens* (PDB code: 3REN) and the PssZ protein from *L. monocytogens* (PDB code: 6R2M) was always recognized as the best structural template for their catalytic (α/α)_6_-barrel domain homology modelling in the ratio 16:1, respectively (not shown).

**Fig. 2:**
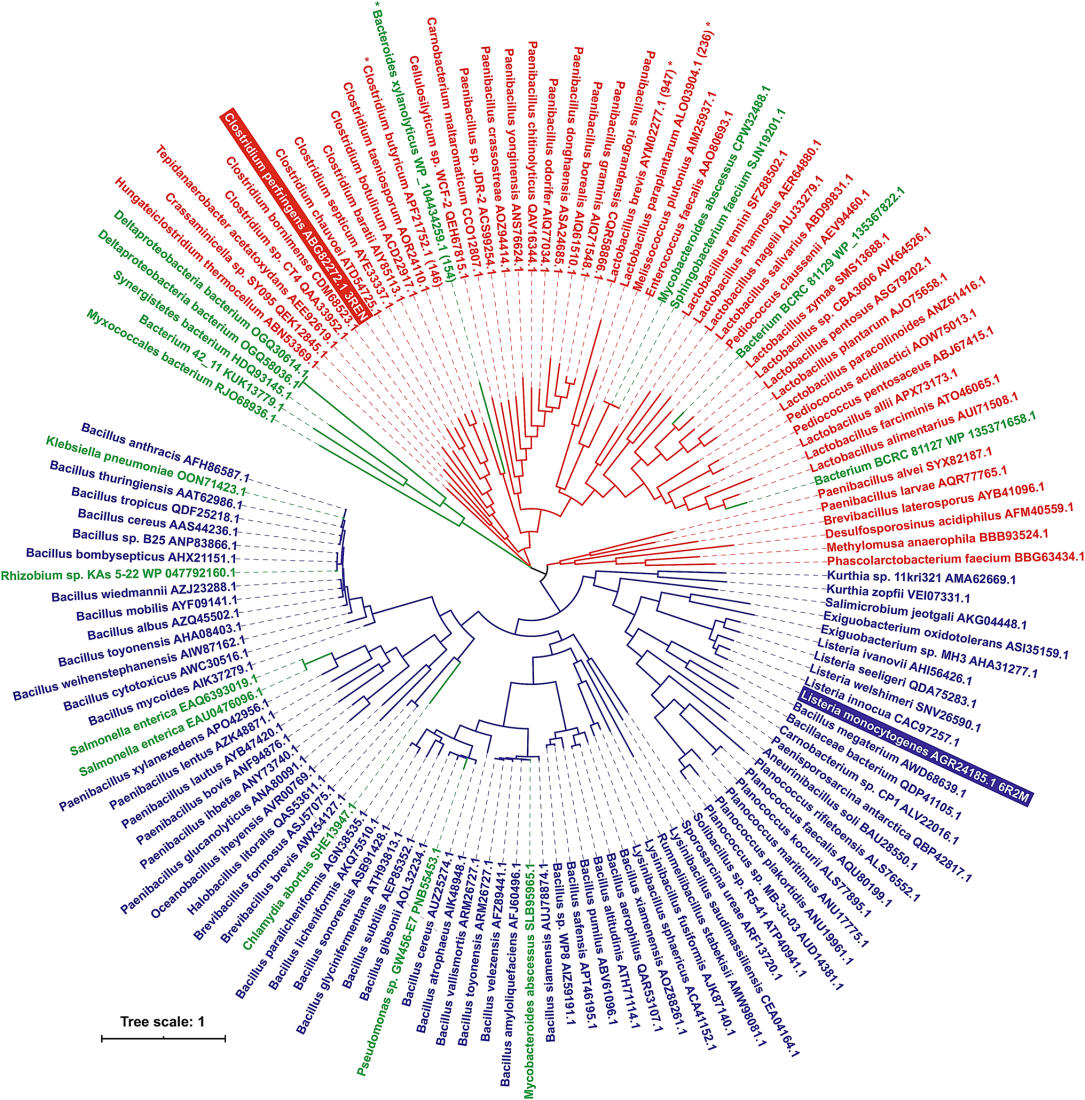
Evolutionary tree of the family GH126. The tree consists of 117 unique non-redundant sequences of the family GH126 (all from Firmicutes) and 17 additional potential family members originating outside the phylum Firmicutes. The tree is based on the alignment of complete sequences (for details, see Fig. S1). The two large evolutionary groups identified previously (Kerenyiova and Janecek 2020) represented by the CPF_2247 amylolytic enzyme from *C. perfringens* (48 members; red colour) and the PssZ protein from *L. monocytogenes* (69 members; blue colour) are completed by additional out-of-Firmicutes sequences coloured green. Each protein is labelled by the name of the organism and the GenBank accession number. Four proteins containing the N-terminal extensions that were cut for making the alignment are marked by an asterisk; the length of the extension being indicated in parentheses. With regard to bootstrap values (not shown to preserve the clarity), they were ≥ 50% for more than 83% of interior branches

To illustrate the evolutionary relationships of the newly identified group of 17 potential out-of-Firmicutes family GH126 members within the family, their sequences were aligned together with 117 already established GH126 members selected previously (Kerenyiova and Janecek 2020). Since the sequence comparison was focused on the catalytic (α/α)_6_-barrel fold, the N-terminal segment (residues 1-154) of the protein from *Bacteroides xylanolyticus* (GenBank accession No. WP_104434259.1) was eliminated, similar to the 3 proteins from the original set of 117 sequences (Fig. S1). The alignment required only a subtle manual adjustment to maximize sequence similarities, especially with regard to seven CSRs, warranting the calculation of two maximum-likelihood evolutionary trees: (1) one based on the alignment of complete sequences (Fig. 2); and (2) the other one based on the alignment of seven selected CSRs (Fig. S2). Although the distribution of individual sequences of both groups—i.e., those from the original set of 117 proteins as well as those from 17 newly identified ones—was found to be not identical in the 2 evolutionary trees, the basic division of 117 established family GH126 members into 2 groups represented by the CPF_2247 amylolytic enzyme from *C. perfringens* (Ficko-Blean et al. 2011) and the PssZ protein from *L. monocytogenes* (Wu et al. 2019), observed previously (Kerenyiova and Janecek 2020), has been preserved (Fig. 2; Fig. S2). As far as the clustering of the 17 new potential family GH126 members is concerned, they have been scattered in both trees without obvious reflecting their bacterial phylum origin. However, in spite of their taxonomically irrespective clustering, no exchange between the two basic groups (*C. perfringens* CPF_2247 and *L. monocytogenes* PssZ proteins) was observed in both evolutionary trees, i.e. in each tree, the same 10 and 7 new proteins were found as follows: (1) in the group of CPF_2247 amylolytic enzyme from *C. perfringens*—Bacterium BCRC 81127 (GenBank accession No.: WP_135371658.1), Bacterium BCRC 81129 (WP_135367822.1), Bacterium 42_11 (KUK13779.1), *Bacteroides xylanolyticus* (WP_104434259.1), *Deltaproteobacteria bacterium* (OGQ30614.1), *Deltaproteobacteria bacterium* (OGQ30614.1), *Mycobacteroides abscessus* (CPW32488.1), *Sphingobacterium faecium* (SJN19201.1), *Synergistetes bacterium* (HDQ93145.1) and *Myxococcales bacterium* (RJO68936.1); and (2) in the group of PssZ protein from *L. monocytogenes*—*Pseudomonas* sp. GW456-E7 (PNB55453.1), *Chlamydia abortus* (SHE13947.1), *Klebsiella pneumonia* (OON71423.1), *Mycobacteroides abscessus* (SLB95965.1), *Rhizobium* sp. KAs 5-22 (WP_047792160.1), *Salmonella enterica* (EAU0476096.1) and *Salmonella enterica* (EAQ6393019.1). Considering the close relationship of the 17 newly identified potential family GH126 members with Firmicutes counterparts, it seems likely that at least some of them could originate by gene acquisition in the process of horizontal gene transfer, a well-known phenomenon precisely documented in the main α-amylase family GH13 (Da Lage et al. 2004, 2013; Chen et al. 2012; Desiderato et al. 2020).

Unfortunately, since of ~1000 sequences classified currently in the family GH126 in the CAZy database (Loimbard et al. 2014) only two have been biochemically characterized—the CPF_2247 protein from *C. perfringens* as a potential α-amylase (Ficko-Blean et al. 2011) and the PssZ protein from *L. monocytogenes* as an exopolysaccharide-specific glycosidase, the exopolysaccharide being composed from the N-acetylmannoseamine and galactose in a ratio 2:1 (Koseoglu et al. 2015; Wu et al. 2019)—right now, it is not possible to draw any relevant conclusions concerning the possible enzyme specificity of the 17 out-of-Firmicutes proteins (Table 1).

### In silico characterization of the family GH126 non-catalytic terminal domains

The absolutely vast majority of the family GH126 members consist of a single domain protein consisting of catalytic (α/α)_6_-barrel fold identified for the family two representatives, the CPF_2247 amylolytic enzyme (Ficko-Blean et al. 2011) and PssZ protein from *L. monocytogenes* (Wu et al. 2019). The detailed inspection of the current family members has revealed that, in fact, only less than 1% of the family—i.e., 9 sequences—contains additional extension either preceding or succeeding the catalytic barrel; the 10th example being found among the 17 newly delivered potential family members originating outside the Firmicutes (Table 2). Overall, of the all ten cases, nine proteins have the N-terminal extension, whereas only one protein (the protein No. 1 in Table 2; GenBank accession No.: QHK13041.1) possesses the extension at its C-terminal end.

To get an idea about the fold and eventual function of those terminal extensions, the sequences of all ten proteins mentioned above were submitted to the Phyre2 server for their fold recognition and homology modelling. Concerning the N-terminus, the results have revealed the presence of two types of a conserved domain—the thioredoxin-like fold (four cases) and the so-called leucine-rich repeat (LRR) motif (four cases), while a diguanylate cyclase domain containing the GGDEF motif has been identified in the single protein with the C-terminal extension (Table 2). Note, that in one case, the protein from *Heliorestis convoluta* (GenBank accession No.: QGG46501.1), no relevant conserved tertiary structure has been recognized in its N-terminal extension. It is worth mentioning that the modular building of these family GH126 members (which all are supposed to be glycoside hydrolases) with additional domains can remind of proteins evolved using domain shuffling or horizontal domain transfer, seen particularly in the case of starch-binding domains that usually preserve the basic features of their function (Janecek et al. 2019).

Most of carbohydrate-active enzymes are modular proteins possessing, in addition to their catalytic domain, also some extra modules (Lombard et al. 2014). Of these, the best known non-catalytic modules may be represented by CBMs (Boraston et al. 2004; Armenta et al. 2017). Since the family GH126 could be another α-amylase family in the system of CAZy classification (Janecek et al. 2014; Kerenyiova and Janecek 2020), it could be reasonable to look for the presence of some kind of SBDs that have been currently classified in 15 different CBM families in CAZy (Janecek et al. 2019). It is, however, worth mentioning that none of the extensions of sequences from the family GH126 studied here was recognized to contain either an SBD, or a CBM in general (Table 2). The same applies, i.e. no presence for the other two domains and/or motifs—the S-layer-like homology and the fibronectin type-III domain—that are also well distributed in sequences of several GH families (Zona and Janecek 2005; Valk et al. 2017).

The results achieved by homology modelling were verified by submitting the sequence data to CDD and Pfam databases. The correctness of conserved domains identified by the Phyre2 server were confirmed in each of the ten cases by at least one of the two databases mentioned above, or in most cases by both the CDD and Pfam (Table 2).

**Fig. 3:**
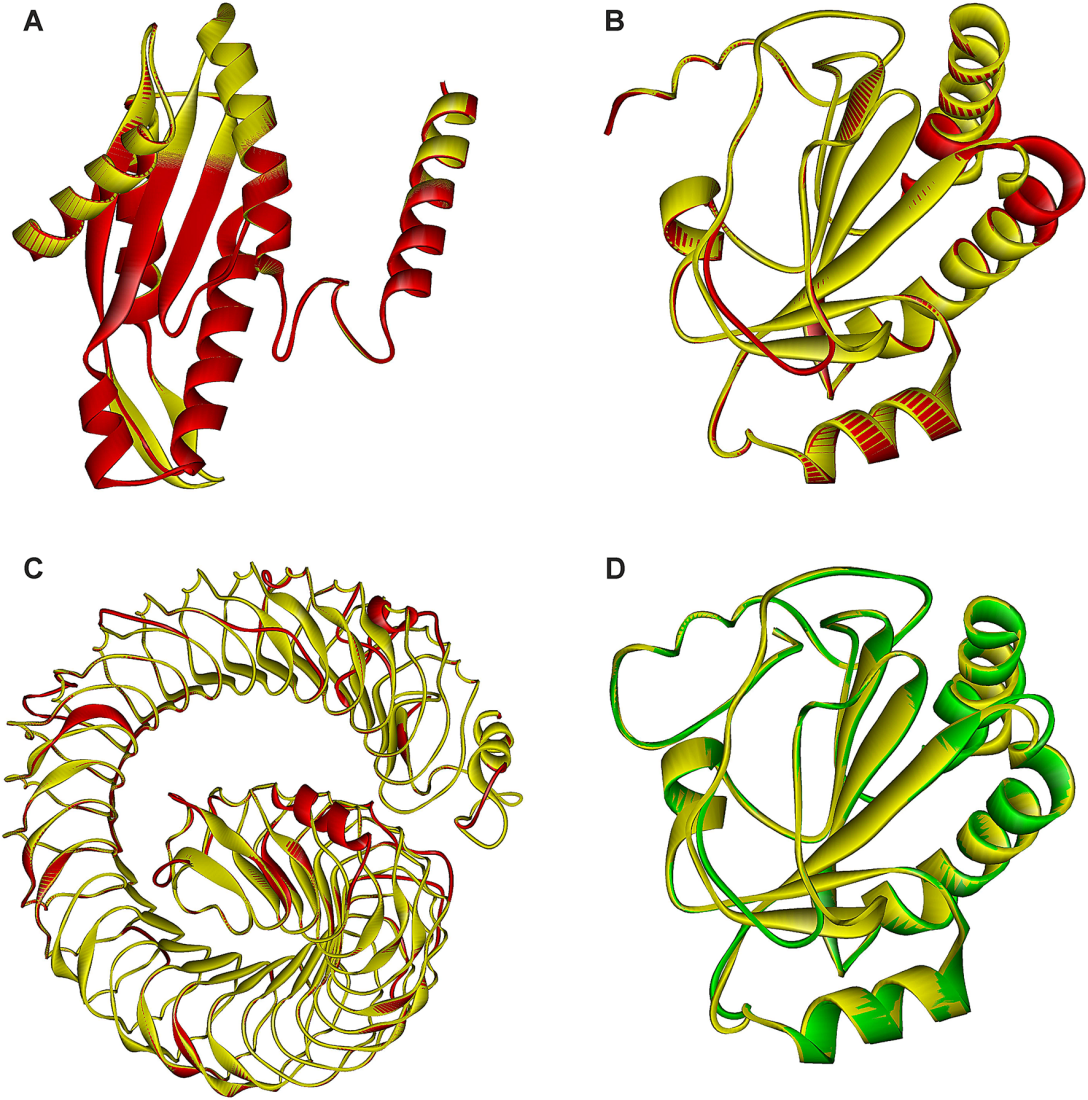
Structural models of terminal extra domains of family GH126 members. **a** The model of the C-terminal extension of the protein from *Bacillus velezensis* (GenBank accession No.: QHK13041.1; residues S458-E636; red) overlapped with the corresponding part of a signalling protein from *Caulobacter vibrioides* (PDB code: 1W25; residues L261-K442; yellow); **b** the model of the N-terminal extension of the protein from *Clostridium butyricum* (AXB84457.1; residues I41-S207; red) overlapped with the thioredoxin-like fold present in the protein Rv2874 from *Mycobacterium tuberculosis* (2HYX; residues I376-K545; yellow); **c** the model of the N-terminal extension of the protein from *Lactobacillus brevis* (AYM02277.1; residues S37-G759; red) with the leucine-rich-repeat domain present in the Ser/Thr-protein kinase from *Arabidopsis thaliana* (6S6Q; residues T29-N-859; yellow); and **d** the model of the N-terminal extension of the protein from *Bacteroides xylanolyticus* (WP_104434259.1; residues N37-E209; green) overlapped with the thioredoxin-like fold present in the protein Rv2874 from *Mycobacterium tuberculosis* (2HYX; residues E366-N542; yellow). The individual superimposed parts cover: **a** 179 Cα-atoms with a 0.24 Å RMSD; **b** 162 Cα-atoms with a 0.50 Å RMSD; **c** 676 Cα-atoms with a 0.59 Å RMSD; and **d** 170 Cα-atoms with a 0.57 Å RMSD. Note, all templates are in each case coloured yellow, whereas the models are shown in red (**a**, **b** and **c**) or green (**d**) depending on the fact whether or not the protein has already been classified in the family GH126

Figure 3 thus illustrates the three examples of extra domains identified in ten proteins from the family GH126 (Table 2): (1) the model of the diguanylate cyclase domain found in the C-terminal extension of the protein from *Bacillus velezensis* (GenBank accession No.: QHK13041.1) (Fig. 3a); (2) the models of the thioredoxin-like fold present in the proteins from *Clostridium butyricum* (AXB84457.1) and *Bacteroides xylanolyticus* (WP_104434259.1) (Fig. 3b,d); and (3) the model of the LRR motif recognized in the protein from *Lactobacillus brevis* (AYM02277.1) (Fig. 3c). Note that the thioredoxin-like fold analogous to that shown for *C. butyricum* and *B. xylanolyticus* proteins (Fig. 3b,d) has been seen also in the N-terminal extensions of the two more proteins from *C. butyricum* (GenBank accession Nos.: QJU43754.1 and APF21752.1; Table 2). As far as the LRR motif is concerned, only the one found in the *L. brevis* protein has been more than 700 residues long (Fig. 3c); the LRR models of three additional proteins from *Lactobacillus bifermentas* (QGG60425.1), *Lactobacillus paraplantarum* (ALO03904.1) and *Lactobacillus* sp. CBA3606 (AVK64614.1) have been substantially shorter (Table 2).

With regard to the structure of a diguanyl cyclase domain positioned C-terminally in the *B. velezensis* GH126 protein (Fig. 3a), it was modelled according to that domain present in a signalling protein PleD, which is the unorthodox response regulator from *Caulobacter vibrioides* (Chan et al. 2004). Diguanylate cyclase usually contains a characteristic GGDEF sequence motif (Galperin et al. 2001). In PleD, it represents the catalytic domain formed by a five-stranded central β-sheet surrounded by helices with the specific motif 368_GGEEF, the Glu371 being involved in catalysis (Chan et al. 2004). In the model of the C-terminal domain of the family GH126 protein from *B. velezensis* (Fig. 3a), the region 565_SAERF corresponds with 368_GGEEF, i.e. an Arg568 occupies the position of the functional Glu371 from PleD, indicating the original role could hardly be preserved.

The other conserved domain, the thioredoxin-like fold—identified in the N-terminal extensions of the three GH126 family members from *C. butyricum* as well as of the newly found potential GH126 member from *B. xylanolyticus* (Table 2)—was best modelled according to the template thioredoxin-like domain of the C-terminal ectodomain of electron transporter Rv2874 (protein DipZ) from *Mycobacterium tuberculosis* (Goldstone et al. 2016). Typical thioredoxin fold consists of four β-strands surrounded by three α-helices (Pan and Bardwell 2006), the motif being clearly seen in both selected family GH126 members (Fig. 3b, d). Interestingly, in the Rv2874 protein, which is responsible for correctly formed disulphide bonds in secreted or surface-associated proteins from *M. tuberculosis*, the entire C-terminal ectodomain is formed by the N-terminally positioned thioredoxin-like fold succeeded by a cellulose binding CBM (Goldstone et al. 2016). One of the best known features of a thioredoxin fold is the presence of a Cys-X-X-Cys motif in the active site (Pan and Bardwell 2006). While the Rv2874 motif Cys437-Ile-Asn-Cys440 (Goldstone et al. 2016) has no correspondence in any of the three GH126 proteins from *C. butyricum*, the equivalent motif Cys103-Pro-Asp-Cys106 is present in the potential family GH126 member from *B. xylanolyticus* (not shown) indicating the function might have been preserved in this protein. The two further reliable structural templates for the thioredoxin-like fold in the family GH126 members have been identified in the human NHL repeat-containing protein 2 (Biterova et al. 2018) and the mouse selenocysteine-dependent iodothyronine deiodinase (Schweizer et al. 2014).

As far as the very long N-terminal extension of the GH126 protein from *L. brevis* is concerned, it was convincingly modelled as a LRR (Fig. 3c) present in various receptor Ser/Thr-protein kinases from *Arabidopsis thaliana*, such as GSO1 (Okuda et al. 2020), FLG22 (Sun et al. 2013) and PEPR1 (Tang et al. 2015), which are used to sense peptide hormones with diverse sequences at the cell surface. They belong to the LRR receptor kinase family of membrane integral receptors counting in *Arabidopsis* more than 200 members (Chakraborty et al. 2019). A typical LRR was originally recognized as a structural motif consisting of repetitive regions of 20–30 amino acid residues rich in leucine, the tandem repeats being connected together forming a solenoid shape (Kobe and Deisenhofer 1994; Enkhbayar et al. 2003). Although the analysed N-terminal extension of the family GH126 protein from *L. brevis* seems to be long enough to adapt an active LRR fold (Fig. 3c), the fact that its template LRR motifs are present in various members of plant LRR receptor kinase family (Chakraborty et al. 2019) currently precludes to make a more conclusive prediction concerning its exact function. Finally, concerning the N-terminal extensions of the GH126 proteins from the remaining three lactobacilli (Table 2), approximately 100-residue long segment from those extensions could contain the N-terminal part of the homologue of LRR motif present in various virulence factors called internalins from *Listeria monocytogenes* (Ooi et al. 2006; Bublitz et al. 2008; Neves et al. 2013).

## Conclusions

The present bioinformatics study was undertaken with the main goal to extend the taxonomic scope of the family GH126 since until now, only proteins from bacterial phylum Firmicutes have been officially classified into the family. BLAST searches using the two characterized family GH126 members as queries, i.e., the CPF_2247 amylolytic enzyme from *C. perfringens* and the PssZ protein from *L. monocytogenes*, have revealed 17 proteins outside Firmicutes exhibiting clear sequence-structural features characteristic of the family, including the potential catalytic machinery, important conserved residues as well as seven typical CSRs. The additional aim of the present study was to characterize the N- and C-terminal extensions present in ten family GH126 members (i.e. nine current members and one new potential one) by structure homology modelling. The results of the Phyre2 server have recognized the well-conserved LRR motifs and the thioredoxin-like fold positioned N-terminally in eight family GH126 cases, whereas a diguanylate cyclase domain with characteristic GGDEF motif has been identified in one protein possessing the C-terminal extension.

## Electronic supplementary material

Below is the link to the electronic supplementary material.

Supplementary file1 (EPS 28160 kb) Fig. S1 Sequence alignment of selected family GH126 members. The two large evolutionary groups identified previously (Kerenyiova and Janecek 2020) – 117 proteins together – represented by the CPF_2247 amylolytic enzyme from C. perfringens (48 members; red colour) and the PssZ protein from L. monocytogenes (69 members; blue colour) are completed by additional 17 out-of-Firmicutes proteins coloured green. The alignment covers the entire sequences, except for the four sequences as follows: (i) No. 17 – Clostridium butyricum (GenBank accession No. APF21752.1; residues 1-146); (ii) No. 18 – Bacteroides xylanolyticus (WP_104434259.1; 1-154) (iii) No. 30 – Lactobacillus brevis (AYM02277.1; 1-947); and (iv) No. 31 – Lactobacillus paraplantarum (ALO03904.1; 1-236). The seven proposed CSRs are boxed. The two potential catalytic residues – Glu84 in CSR-1 and Asp136 in CSR-3 (CPF_2247 numbering) as well as the potentially functional aromatics – Tyr194 in CSR-5 are italicized and signified by asterisks under the alignment. The ordering of sequences from the top corresponds with their appearance in the evolutionary tree (Fig. 1) that was calculated based on this alignment. The colour code for the selected residues: W, yellow; F, Y – blue; V, L, I – green; D, E – red; R, K – cyan; H – brown; C – magenta; G, P – black.

Supplementary file2 (EPS 5233 kb) Fig. S2 Evolutionary tree of the family GH126 based on the alignment of 7 conserved sequence regions. The tree consists of 117 unique non-redundant sequences of the family GH126 (all from Firmicutes) and 17 additional potential family members originating outside the phylum Firmicutes. The two large evolutionary groups identified previously (Kerenyiova and Janecek 2020) represented by the CPF_2247 amylolytic enzyme from C. perfringens (48 members; red colour) and the PssZ protein from L. monocytogenes (69 members; blue colour) are completed by additional out-of-Firmicutes sequences coloured green. Each protein is labelled by the name of the organism and the GenBank accession number. Four proteins containing the N-terminal extensions that were cut for making the alignment are marked by an asterisk; the length of the extension being indicated in parentheses. With regard to bootstrap values (not shown in order to preserve the clarity), they were ≥50% for almost 50% of interior branches.

## Abbreviations

CAZy: Carbohydrate-active enzymes
CBM: Carbohydrate-binding module
CDD: Conserved Domain Database
CSR: Conserved sequence region
GH: Glycoside hydrolase
LRR: Leucine-rich repeat
PDB: Protein Data Bank
SBD: Starch-binding domain
RMSD: Root-mean square deviation
